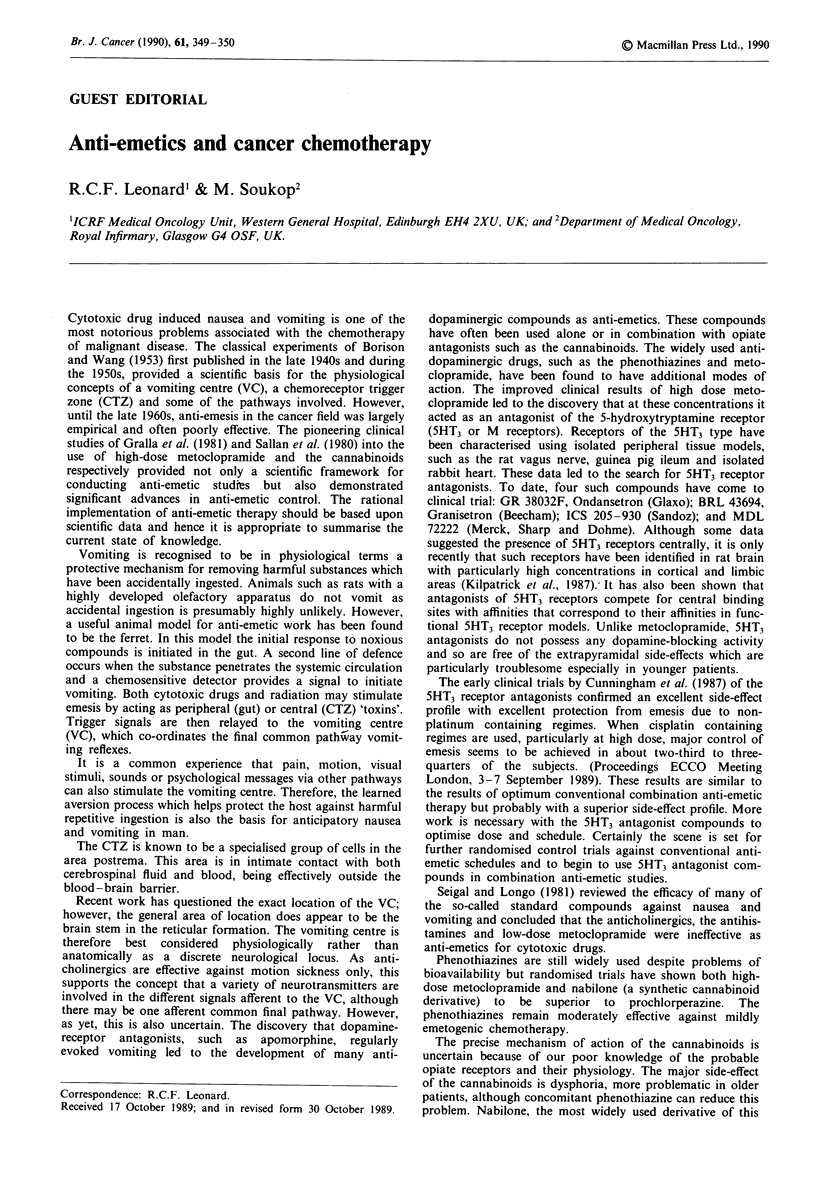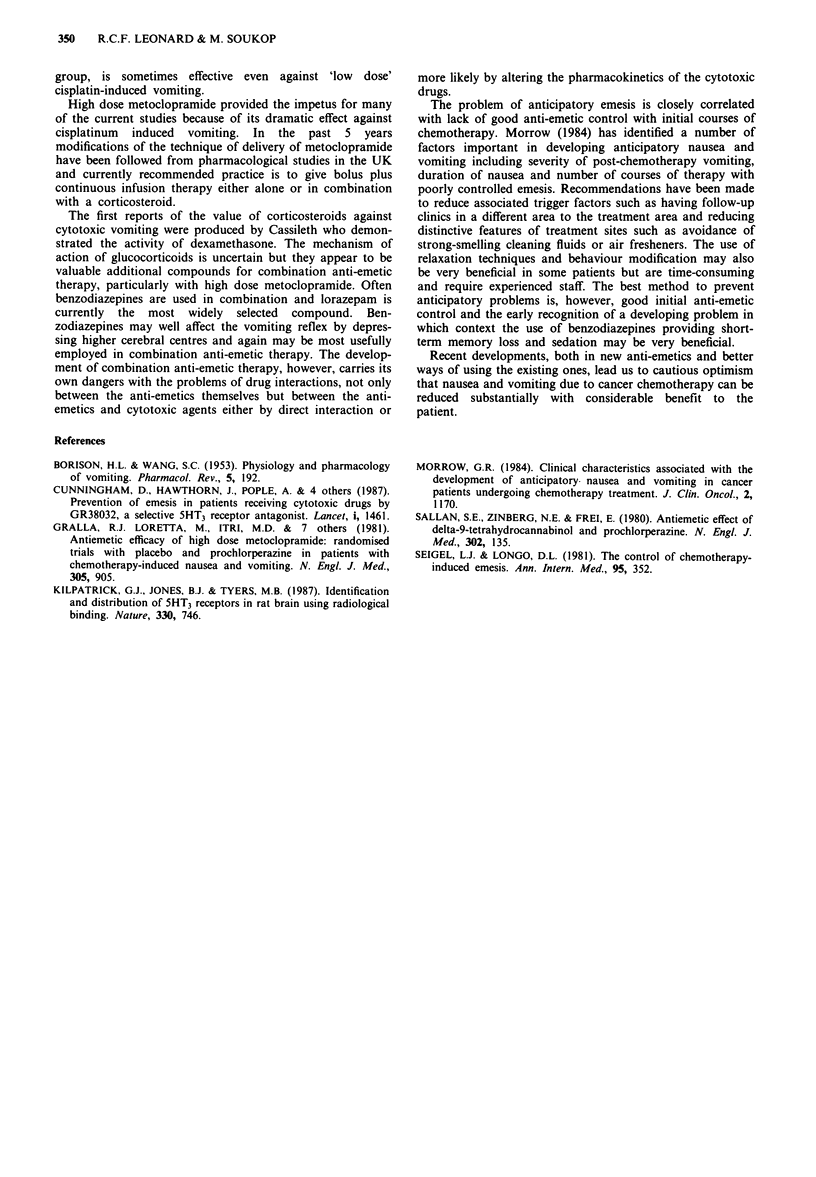# Anti-emetics and cancer chemotherapy.

**DOI:** 10.1038/bjc.1990.76

**Published:** 1990-03

**Authors:** R. C. Leonard, M. Soukop

**Affiliations:** ICRF Medical Oncology Unit, Western General Hospital, Edinburgh, UK.


Br. J. Cancer (1990), 61, 349–350 © Macmillan Press Ltd., 1990

GUEST EDITORIAL

### Anti-emetics and cancer chemotherapy

R.C.F. Leonard[1] & M. Soukop[2]

[1]ICRF Medical Oncology Unit, Western General Hospital, Edinburgh EH4 2XU, UK; and [2]Department of Medical Oncology, Royal Infirmary, Glasgow G4 OSF, UK.

Cytotoxic drug induced nausea and vomiting is one of the most notorious problems associated with the chemotherapy of malignant disease. The classical experiments of Borison and Wang (1953) first published in the late 1940s and during the 1950s, provided a scientific basis for the physiological concepts of a vomiting centre (VC), a chemoreceptor trigger zone (CTZ) and some of the pathways involved. However, until the late 1960s, anti-emesis in the cancer field was largely empirical and often poorly effective. The pioneering clinical studies of Gralla *et al.* (1981) and Sallan *et al.* (1980) into the use of high-dose metoclopramide and the cannabinoids respectively provided not only a scientific framework for conducting anti-emetic studies but also demonstrated significant advances in anti-emetic control. The rational implementation of anti-emetic therapy should be based upon scientific data and hence it is appropriate to summarise the current state of knowledge.

Vomiting is recognised to be in physiological terms a protective mechanism for removing harmful substances which have been accidentally ingested. Animals such as rats with a highly developed olefactory apparatus do not vomit as accidental ingestion is presumably highly unlikely. However, a useful animal model for anti-emetic work has been found to be the ferret. In this model the initial response to noxious compounds is initiated in the gut. A second line of defence occurs when the substance penetrates the systemic circulation and a chemosensitive detector provides a signal to initiate vomiting. Both cytotoxic drugs and radiation may stimulate emesis by acting as peripheral (gut) or central (CTZ) 'toxins'. Trigger signals are then relayed to the vomiting centre (VC), which co-ordinates the final common pathway vomiting reflexes.

It is a common experience that pain, motion, visual stimuli, sounds or psychological messages via other pathways can also stimulate the vomiting centre. Therefore, the learned aversion process which helps protect the host against harmful repetitive ingestion is also the basis for anticipatory nausea and vomiting in man.

The CTZ is known to be a specialised group of cells in the area postrema. This area is in intimate contact with both cerebrospinal fluid and blood, being effectively outside the blood–brain barrier.

Recent work has questioned the exact location of the VC; however, the general area of location does appear to be the brain stem in the reticular formation. The vomiting centre is therefore best considered physiologically rather than anatomically as a discrete neurological locus. As anticholinergics are effective against motion sickness only, this supports the concept that a variety of neurotransmitters are involved in the different signals afferent to the VC, although there may be one afferent common final pathway. However, as yet, this is also uncertain. The discovery that dopamine-receptor antagonists, such as apomorphine, regularly evoked vomiting led to the development of many anti-dopaminergic compounds as anti-emetics. These compounds have often been used alone or in combination with opiate antagonists such as the cannabinoids. The widely used anti-dopaminergic drugs, such as the phenothiazines and metoclopramide, have been found to have additional modes of action. The improved clinical results of high dose metoclopramide led to the discovery that at these concentrations it acted as an antagonist of the 5-hydroxytryptamine receptor ($5HT_3$ or M receptors). Receptors of the $5HT_3$ type have been characterised using isolated peripheral tissue models, such as the rat vagus nerve, guinea pig ileum and isolated rabbit heart. These data led to the search for $5HT_3$ receptor antagonists. To date, four such compounds have come to clinical trial: GR 38032F, Ondansetron (Glaxo); BRL 43694, Granisetron (Beecham); ICS 205–930 (Sandoz); and MDL 72222 (Merck, Sharp and Dohme). Although some data suggested the presence of $5HT_3$ receptors centrally, it is only recently that such receptors have been identified in rat brain with particularly high concentrations in cortical and limbic areas (Kilpatrick *et al.*, 1987). It has also been shown that antagonists of $5HT_3$ receptors compete for central binding sites with affinities that correspond to their affinities in functional $5HT_3$ receptor models. Unlike metoclopramide, $5HT_3$ antagonists do not possess any dopamine-blocking activity and so are free of the extrapyramidal side-effects which are particularly troublesome especially in younger patients.

The early clinical trials by Cunningham *et al.* (1987) of the $5HT_3$ receptor antagonists confirmed an excellent side-effect profile with excellent protection from emesis due to non-platinum containing regimes. When cisplatin containing regimes are used, particularly at high dose, major control of emesis seems to be achieved in about two-third to three-quarters of the subjects. (Proceedings ECCO Meeting London, 3–7 September 1989). These results are similar to the results of optimum conventional combination anti-emetic therapy but probably with a superior side-effect profile. More work is necessary with the $5HT_3$ antagonist compounds to optimise dose and schedule. Certainly the scene is set for further randomised control trials against conventional anti-emetic schedules and to begin to use $5HT_3$ antagonist compounds in combination anti-emetic studies.

Seigal and Longo (1981) reviewed the efficacy of many of the so-called standard compounds against nausea and vomiting and concluded that the anticholinergics, the antihistamines and low-dose metoclopramide were ineffective as anti-emetics for cytotoxic drugs.

Phenothiazines are still widely used despite problems of bioavailability but randomised trials have shown both high-dose metoclopramide and nabilone (a synthetic cannabinoid derivative) to be superior to prochlorperazine. The phenothiazines remain moderately effective against mildly emetogenic chemotherapy.

The precise mechanism of action of the cannabinoids is uncertain because of our poor knowledge of the probable opiate receptors and their physiology. The major side-effect of the cannabinoids is dysphoria, more problematic in older patients, although concomitant phenothiazine can reduce this problem. Nabilone, the most widely used derivative of this

Correspondence: R.C.F. Leonard.
Received 17 October 1989; and in revised form 30 October 1989.

group, is sometimes effective even against 'low dose' cisplatin-induced vomiting.

High dose metoclopramide provided the impetus for many of the current studies because of its dramatic effect against cisplatinum induced vomiting. In the past 5 years modifications of the technique of delivery of metoclopramide have been followed from pharmacological studies in the UK and currently recommended practice is to give bolus plus continuous infusion therapy either alone or in combination with a corticosteroid.

The first reports of the value of corticosteroids against cytotoxic vomiting were produced by Cassileth who demonstrated the activity of dexamethasone. The mechanism of action of glucocorticoids is uncertain but they appear to be valuable additional compounds for combination anti-emetic therapy, particularly with high dose metoclopramide. Often benzodiazepines are used in combination and lorazepam is currently the most widely selected compound. Benzodiazepines may well affect the vomiting reflex by depressing higher cerebral centres and again may be most usefully employed in combination anti-emetic therapy. The development of combination anti-emetic therapy, however, carries its own dangers with the problems of drug interactions, not only between the anti-emetics themselves but between the anti-emetics and cytotoxic agents either by direct interaction or more likely by altering the pharmacokinetics of the cytotoxic drugs.

The problem of anticipatory emesis is closely correlated with lack of good anti-emetic control with initial courses of chemotherapy. Morrow (1984) has identified a number of factors important in developing anticipatory nausea and vomiting including severity of post-chemotherapy vomiting, duration of nausea and number of courses of therapy with poorly controlled emesis. Recommendations have been made to reduce associated trigger factors such as having follow-up clinics in a different area to the treatment area and reducing distinctive features of treatment sites such as avoidance of strong-smelling cleaning fluids or air fresheners. The use of relaxation techniques and behaviour modification may also be very beneficial in some patients but are time-consuming and require experienced staff. The best method to prevent anticipatory problems is, however, good initial anti-emetic control and the early recognition of a developing problem in which context the use of benzodiazepines providing short-term memory loss and sedation may be very beneficial.

Recent developments, both in new anti-emetics and better ways of using the existing ones, lead us to cautious optimism that nausea and vomiting due to cancer chemotherapy can be reduced substantially with considerable benefit to the patient.

### References

BORISON, H.L. & WANG, S.C. (1953). Physiology and pharmacology of vomiting. *Pharmacol. Rev.*, **5**, 192.

CUNNINGHAM, D., HAWTHORN, J., POPLE, A. & 4 others (1987). Prevention of emesis in patients receiving cytotoxic drugs by GR38032, a selective $5HT_3$ receptor antagonist. *Lancet*, **i**, 1461.

GRALLA, R.J. LORETTA, M., ITRI, M.D. & 7 others (1981). Antiemetic efficacy of high dose metoclopramide: randomised trials with placebo and prochlorperazine in patients with chemotherapy-induced nausea and vomiting. *N. Engl. J. Med.*, **305**, 905.

KILPATRICK, G.J., JONES, B.J. & TYERS, M.B. (1987). Identification and distribution of $5HT_3$ receptors in rat brain using radiological binding. *Nature*, **330**, 746.

MORROW, G.R. (1984). Clinical characteristics associated with the development of anticipatory nausea and vomiting in cancer patients undergoing chemotherapy treatment. *J. Clin. Oncol.*, **2**, 1170.

SALLAN, S.E., ZINBERG, N.E. & FREI, E. (1980). Antiemetic effect of delta-9-tetrahydrocannabinol and prochlorperazine. *N. Engl. J. Med.*, **302**, 135.

SEIGEL, L.J. & LONGO, D.L. (1981). The control of chemotherapy-induced emesis. *Ann. Intern. Med.*, **95**, 352.